# Primary hepatic epithelioid hemangioendothelioma: a case report

**DOI:** 10.1177/03000605241306649

**Published:** 2024-12-28

**Authors:** Jing-Rui Wang, Qi-Jun Yang, Bei Lu, Yang Cai, Jun-Jie Yin

**Affiliations:** 1 Department of Hepatobiliary Surgery, Affiliated Hangzhou First People’s Hospital, Westlake University, School of Medicine, Hangzhou, China

**Keywords:** Hepatic epithelioid hemangioendothelioma, transcatheter arterial chemoembolization, liver transplantation, anlotinib, metastasis, glucose metabolism

## Abstract

Epithelioid hemangioendothelioma is a low-grade malignant tumor of vascular origin. The rarity of hepatic epithelioid hemangioendothelioma (HEHE) makes the diagnosis and treatment of this entity challenging. We report a case of a 69-year-old female patient who suffered from HEHE and complained of abdominal distension pain with dizziness and appetite loss for more than half a month. Enhanced computed tomography of the upper abdomen indicated multiple space-occupying lesions in the liver. The pathological results of color ultrasound puncture suggested HEHE. We performed transcatheter arterial chemoembolization and relevant examinations according to the patient’s condition and their choice. We followed the patient for 5 years and found that she developed recurrent intrahepatic metastasis of the tumor. Computed tomography was performed again after 3 months of treatment with anlotinib and the tumor did not show any progression. HEHE is a relatively rare hepatic malignant tumor derived from vascular endothelial cells, with a low incidence, atypical clinical manifestations, and a difficult diagnosis that can only be confirmed with pathological results. Currently, appropriate treatment methods should be selected according to the specific conditions of the patient.

## Background

Epithelioid hemangioendothelioma is a rare malignant tumor that originates from vascular endothelial cells. This tumor can occur in all parts of the body, mostly in the soft tissues of the limbs, and can also occur in other organs, such as the lungs, bone, spleen, and brain.^1^ Hepatic epithelioid hemangioendothelioma (HEHE) is rare, with an incidence of approximately 0.1 in 100,000.^2^ The median overall survival of HEHE is 16.9 years, with an overall survival of 89% at 1 year, 68% at 5 years, and 64% at 10 years.^3^ The etiology of HEHE remains unclear. The possible etiological factors of HEHE include oral contraceptives, progesterone imbalance, liver injury, alcohol, chloroethylene pollution, viral hepatitis, cirrhosis, and long-term use of immunosuppressive agents after liver transplantation.^4^

The diagnosis of HEHE is mainly based on pathology. Microscopically, the tumor cells are mostly arranged in a dense and disordered pattern, with a cord-like or nested cord-like distribution, and the cellular morphology is mostly epithelioid, fusiform, or irregular. Additionally, there is hypertrophy of irregular nuclei of tumor cells, with uneven chromatin or a coarse granular appearance, and positivity of CD34, CA31, ERG, and Friend leukemia virus integration 1 (FLI-1).^5^ Surgical resection has been reported to be an effective treatment for HEHE.^6^ Transcatheter arterial chemoembolization (TACE), liver transplantation, chemotherapy, targeted therapy, and immunotherapy have been used in the treatment of patients with HEHE.^5–7^

We report an elderly female patient with HEHE who was treated by TACE and anlotinib.

## Case presentation

A 69-year-old woman complained of abdominal distension pain with dizziness and appetite loss for more than half a month. Before half a month, without obvious inducement, she developed upper abdominal pain, which was gradually relieved after rest, with jaundice, loss of appetite, and fatigue. There were no major comorbidities at admission. The patient was a non-smoker, without a personal or family history of other diseases.

A physical examination upon admission showed the following: jaundice, upper abdominal pain, no palmar erythema, no spider angioma, a 7-cm old surgical scar in the right abdomen, no Murphy’s sign, and no shifting dullness. Laboratory examinations showed that serum α-fetoprotein, carcinoembryonic antigen, and carbohydrate antigen 199 values were within normal limits. Liver function, renal function, electrolytes, coagulation function, routine blood parameters, routine urine parameters, and qualitative analysis of hepatitis B, hepatitis A virus-immunoglobulin (Ig) M/IgG, hepatitis C virus-IgG, hepatitis D virus-IgG, and hepatitis E virus-IgG were normal.

**Figure 1.**
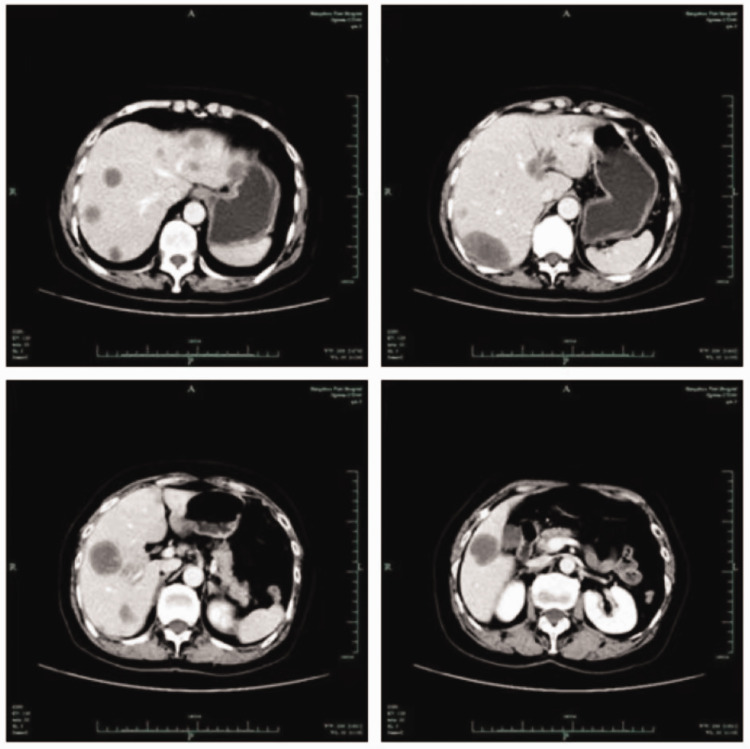
Enhanced computed tomography showing multiple liver space-occupying lesions.

**Figure 2.**
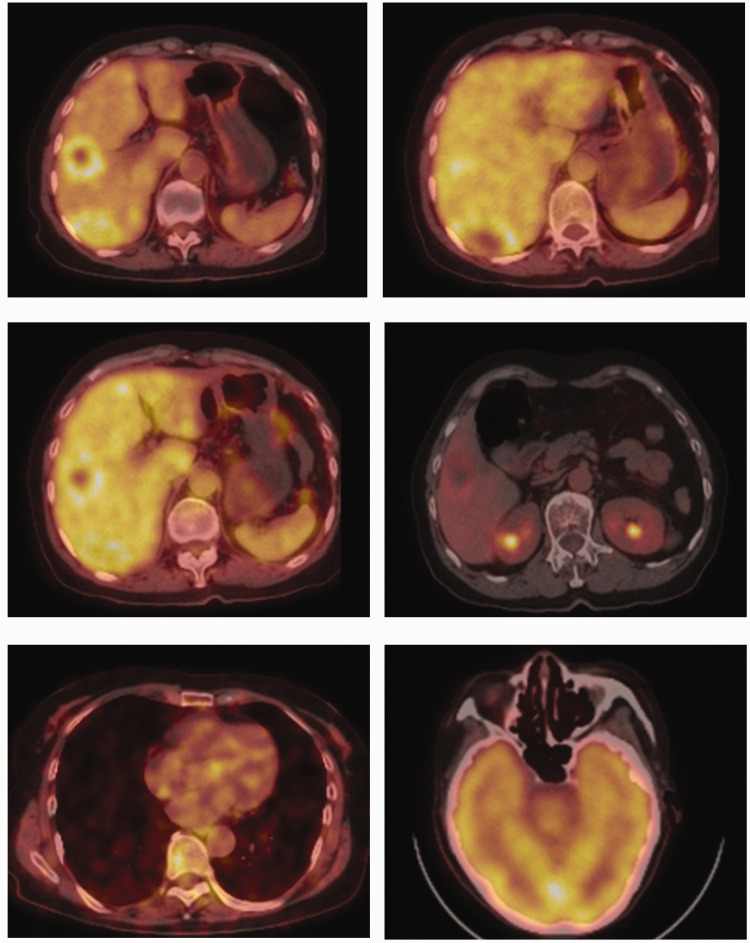
Positron emission tomography-computed tomography shows multiple intrahepatic space-occupying lesions and increased glucose metabolism. No increased glucose metabolism was observed in the lungs or skull.

**Figure 3.**
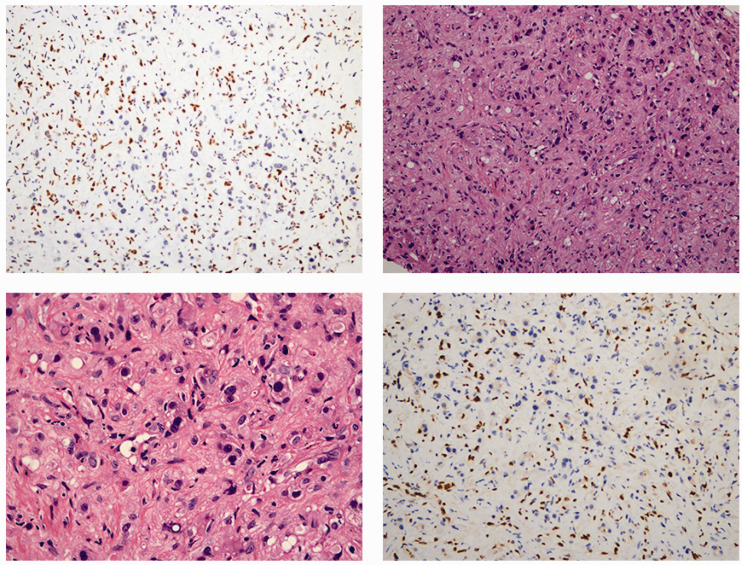
Immunohistochemical findings. Hematoxylin and eosin staining is shown (200×, top right panel; 400×, bottom left panel). Immunohistochemical staining of ERG (top left panel) and Friend leukemia virus integration 1 (bottom right panel) is also shown.

Enhanced computed tomography (CT) of the upper abdomen showed that the surface of the liver was smooth, and multiple circular low-density shadows were observed in the liver (Figure 1). The largest shadow was located in the posterior segment of the right lobe of the liver, with a size of 5.3 × 2.6 cm. A contrast-enhanced scan showed mild circumferential enhancement and intrahepatic bile duct dilation. These CT findings indicated multiple liver space-occupying lesions. A chest high-resolution plain CT scan showed multiple micronodules scattered in both lungs, and metastasis was not excluded. Positron emission tomography-CT showed multiple intrahepatic space-occupying lesions and increased glucose metabolism, and malignant lesions were considered (Figure 2). Additionally, this technique showed multiple nodules in both lungs, with no increase in glucose metabolism, and the nature (benign or malignant) of the lesions remained to be determined. We decided to perform an ultrasound-guided biopsy of the liver, which showed HEHE. Immunohistochemical findings were as follows: vimentin (+) CD31 (+) CD34 (+) F8 (+) epithelial membrane antigen (−), thyroid transcription factor-1 (−), Ki-67 (+, 2% to 5%), ERG (+), and FLI-1 (+) (Figure 3). According to clinical, imaging, and pathological findings, the patient was diagnosed with HEHE. Multiple intrahepatic tumors in this patient could not be radically resected. We performed TACE and CT and positron emission tomography-CT examinations according to the patient’s condition and their choice.

**Figure 4.**
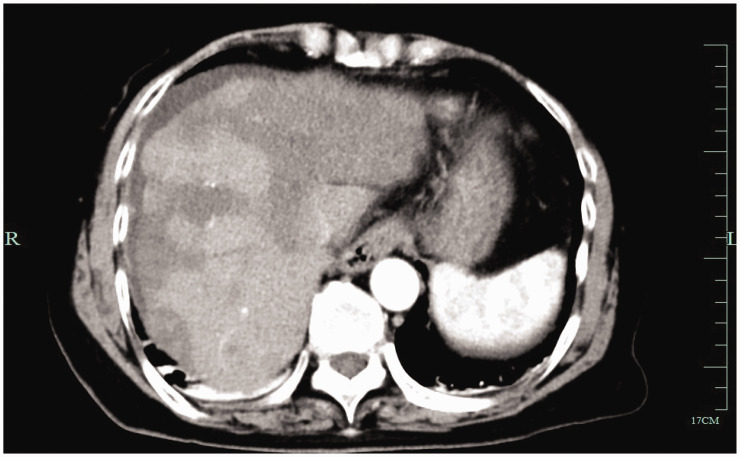
Enhanced computed tomography showing recurrent intrahepatic metastasis of the tumor at a 5-year follow-up.

**Figure 5.**
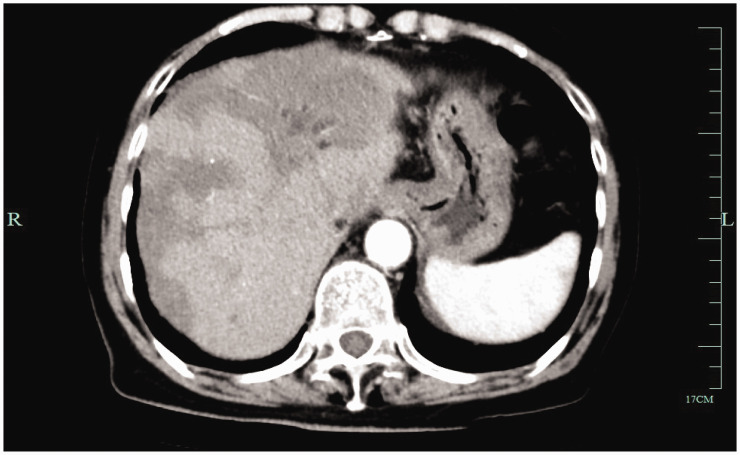
Enhanced computed tomography showing no tumor progression 3 months after starting anlotinib.

We followed the patient for 5 years and found that she developed recurrent intrahepatic metastasis of the tumor (Figure 4). She started treatment with anlotinib, and a CT scan 3 months after starting this treatment showed that the tumor had not progressed (Figure 5). The patient had a good quality of life during the follow-up period, was able to live a normal life, and did not experience any complications or drug side effects. Liver function, including alanine aminotransferase and aspartate aminotransferase concentrations, remained within the normal range. The reporting of this study conforms to the CARE guidelines.^8^

## Discussion

Epithelioid hemangioendothelioma was first reported by Wesis and Enzinger in 1982,^9^ and HEHE was then first described by Ishak in 1984.^10^ The onset of HEHE is relatively insipid, and most of the cases have reached the middle or late stage when this disease is diagnosed. The common symptoms of HEHE are epigastric discomfort or pain, fatigue, and a poor appetite. Occasionally, fever and jaundice are observed. Although HEHE is a low-grade malignant tumor, metastasis occurs in one third of patients because of the rich blood sinus of the liver. Tumor cells are prone to invade the terminal branches of the portal vein, and most commonly migrate to the lungs or the abdominal cavity. Patients with a metastatic tumor can die from liver and respiratory failure. Distinguishing a polycentric origin from metastasis is difficult because it can travel from primary organs to other tissues and organs, and there are also multiple primary lesions at the same time.^11^

HEHE usually involves multiple lesions, and most of them are located under the liver capsule or around the liver. The imaging characteristics of HEHE mainly include capsular retraction, calcifications, halo sign, and target sign.^12^ Most plain CT scans of HEHE are low density, and some lesions show a circular shape with a low density. Enhanced CT scans show progressive enhancement, which is related to the size of the lesions.^13^ Magnetic resonance imaging shows a clear tumor structure, and a plain magnetic resonance imaging scan shows hypointensity on T1-weighted images and hyperintensity on T2-weighted images. Larger lesions (>2 cm) are prone to liquefaction necrosis, and the lesion density or signal is uneven.^14^

The diagnosis of HEHE is mainly based on pathology. The gross appearance of HEHE is mostly nodules with infiltrating growth of grayish-white tough masses. Under a microscope, the tumor cells are mostly arranged in a dense and disordered pattern with a cord-like or nested cord-like distribution, and the cell morphology is mostly epithelioid, fusiform, or irregular. Hypertrophic irregular nuclei, with uneven chromatin or a coarse granular appearance are observed. The cytoplasm is abundant and eosinophilic, and there are often vacuoles containing red blood cells in the cytoplasm. The stroma is rich in collagen and mucous or there is hyaline degeneration. There is strong positivity of CD34, CA31, ERG, and FLI-1.^5^ The immunohistochemical results of our patient showed that vimentin CD31, CD34, F8, Ki-67, ERG, and FLI-1 were positive, and thyroid transcription factor-1 and epithelial membrane antigen were negative, leading to the diagnosis of HEHE.

Liver transplantation and hepatic arterial chemoembolization are a therapeutic option for patients with HEHE. When HEHE is detected at the early stage and is isolated or confined to hepatic segments or lobes, radical resection is the first choice, which usually results in a good prognosis. Liver transplantation is an ideal option for patients without radical resection. Lai et al.^15^ reported 149 patients with HEHE who were registered in the European liver transplantation registration system from November 1984 to May 2014. They found that the 1-, 5-, and 10-year survival rates of these patients after liver transplantation were 88.6%, 79.5%, and 74.4%, and the 1-, 5-, and 10-year disease-free survival rates were 88.7%, 79.4%, and 72.8%, respectively. Our patient had multiple tumors within the liver, which made radical resection unfeasible, and she lacked sufficient financial resources to afford the cost of liver transplantation. Therefore, we opted for TACE treatment. After a 5-year follow-up, the patient had developed recurrent intrahepatic metastases of the tumor. Anlotinib has previously been studied in patients with advanced sarcoma, and showed satisfactory effectiveness with few side effects, ease of use, and good patient acceptance.^16^ A case report also showed that anlotinib resulted in a partial response in a patient with HEHE.^17^ Consequently, we initiated anti-tumor treatment with anlotinib in this patient.

## Conclusion

HEHE is a relatively rare malignant tumor derived from vascular endothelial cells, with a low incidence and atypical clinical manifestations, which results in difficulty of a clear diagnosis. Currently, radical resection is the first choice of treatment for HEHE. However, in many cases, the appropriate treatment should be selected according to the patient’s specific situation.

## Data Availability

All data of the patient in this case report are included in the published article.
